# Effects of Dietary Intervention on Inflammatory Markers in Metabolic Syndrome: A Systematic Review and Meta-Analysis

**DOI:** 10.3389/fnut.2022.846591

**Published:** 2022-03-31

**Authors:** Mengjun Wang, Junliang Liu, Zhao Zhang, Haixiong Zhang, Ning Wang, Xi Chen, Xuemei Han, Qian Lu, Shanshan Chi

**Affiliations:** ^1^Department of Endocrinology, The First Affiliated Hospital of Xi'an Jiaotong University, Xi'an, China; ^2^Department of Endocrinology, 521 Hospital of Norinco Group, Xi'an, China; ^3^Department of Epidemiology and Statistics, School of Public Health, Medical College, Zhejiang University, Hangzhou, China

**Keywords:** diets, inflammatory markers, IL-6, metabolic syndrome, meta-analysis

## Abstract

**Background:**

Dietary interventions may modulate inflammatory indicators, but the correlations between dietary intervention and inflammatory markers in metabolic syndrome (MetS) settings remain opaque.

**Objective:**

To evaluate the effects of dietary intervention on interleukin-1β (IL-1β), interleukin-6 (IL-6), tumor necrosis factor-α (TNF-α), and C-reactive protein (CRP) in patients with MetS by systematic review and meta-analysis.

**Methods:**

Databases, including PubMed, Embase, Cochrane Library, Scopus, and Google scholar, were searched from June 2011 to June 2021 for relevant available articles. Standardized mean difference (SMD) was generated as effect size by meta-analysis for continuous variants, including IL-1β, IL-6, TNF-α, and CRP levels. Then, according to study characteristics by dietary patterns of the intervention, subgroup analyses were performed.

**Results:**

Finally, 13 studies comprising a total of 1,101 participants were included for the meta-analysis. IL-6 levels in dietary patients were significantly lower than controls (SMD = −0.30, 95% CI = −0.55, 0.04, *p* = 0.02, I^2^ = 64%). However, IL-1β, TNF-α, and CRP levels did not change significantly compared with the control group. Sensitivity analyses further yielded similar results.

**Conclusions:**

Dietary intervention may help decrease IL-6 rather than IL-1β, TNF-α, or CRP levels in patients with MetS.

## Introduction

Metabolic syndrome (MetS) has become a global epidemic disease due to population aging and lifestyle changes, including diets (1). The various definition and criteria for identifying MetS (2) includes interrelated factors, such as abdominal obesity, insulin resistance, hyperglycemia, hypertension, and dyslipidemia (low high-density lipoprotein and increased triglyceride) (3). Sub-clinical TH1–lymphocyte-mediated innate and chronic low-grade inflammation might partially account for its occurrence (4). The interleukin-1 (IL-1) family, particularly IL-1β, is a group of cytokines that play a central role in the regulation of responses associated with immune and obesity-associated inflammation (5). IL-6 is a major pro-inflammatory cytokine in chronic inflammation that is closely related to insulin resistance, neurodegeneration, cardiovascular disease (CVD), and malignancy (6). A few pro-inflammatory cytokines [IL-6 and tumor necrosis factor-α (TNF-α)] can promote the upgrade of plasma C-reactive protein (CRP) level (7).

Bad eating habits are the controllable factors accelerating the development of inflammation and related diseases, including MetS (8). According to literature, food can modify inflammatory responses. It is also strongly linked to the pathogenesis of MetS (9). A healthy diet could contribute most to managing obesity and MetS (10). Currently, nutritional epidemiology tends to illustrate the relationship between dietary intervention and inflammatory diseases, but do not demonstrate the exact food species (11).

Although some trials have proved that dietary intervention can reduce the serum level of inflammatory factors, it is still controversial if there is an association between dietary intervention and the serum level of inflammatory factors in patients with MetS.

Hence, in this study, we aimed to perform a systematic review and meta-analysis to investigate the effects of dietary intervention on IL-1β, IL-6, TNF-α, and CRP levels in MetS.

## Methods

The current systematic review and meta-analysis was implemented in accordance with the principles of the Preferred Reporting Items for Systematic Reviews and Meta-Analyses (PRISMA) statement (12).

### Literature Search Strategy

Database, including PubMed, Embase, Cochrane Library, Scopus, and Google scholar, were searched from June 15, 2011 to June 15, 2021. We used the following mesh terms: “inflammatory markers,” “diet” in combination with “metabolic syndrome.”

### Study Selection

Inclusion criteria were as follows:

1. Randomized controlled trial (RCT) studies;
2. Conducted dietary intervention/s of more than 4 weeks;
3. Meets the diagnostic criteria of MetS;
4. Studies that provided numbers, means, and standard difference (SDs) of IL-1β, IL-6, TNF-α, and CRP.

Exclusion criteria were as follows:

1. literature reviews, *in vitro* study, animal study, or case report;
2. Included patients who had co-morbidities other than MetS;
3. (Patients were administrated drugs which might change the levels of cytokines of interest;
4. No full text.

### Data Extraction

The following information were collected: (1) publication data (first author's name, publication year, and country), (2) study design, (3) total number of participants, their age, sex, BMI, and study duration, (4) glucose, insulin, blood lipid levels, and blood pressure status, and (5) mean and standard difference for IL-1β, IL-6, TNF-α, and CRP levels.

### Quality Assessment

Qualities of enrolled studies were assessed according to the Cochrane Risk of Bias Tool (13). Two investigators independently assessed the quality and extracted data of all included studies. Any discrepancy was adjudicated by a senior investigator.

### Statistical Analysis

Review Manager 5.3 was implemented in our analyses. *p* < .05 was considered to be statistically significant. Standardized mean difference (SMD) was generated as effect size by meta-analysis for continuous variants, including IL-1β, IL-6, TNF-α, and CRP levels. If I^2^ <50% and *p* > 0.01, a fixed-effect model would be used. Otherwise, a random effect model would be implemented. If I^2^ > 75%, further analysis encompassing sensitive analysis, subgroup analysis, or meta-regression was carried out to explore the source of heterogeneity. Publication bias was evaluated by funnel plot and Egger's tests.

## Results

### Search Results and Characteristics

**Figure 1:**
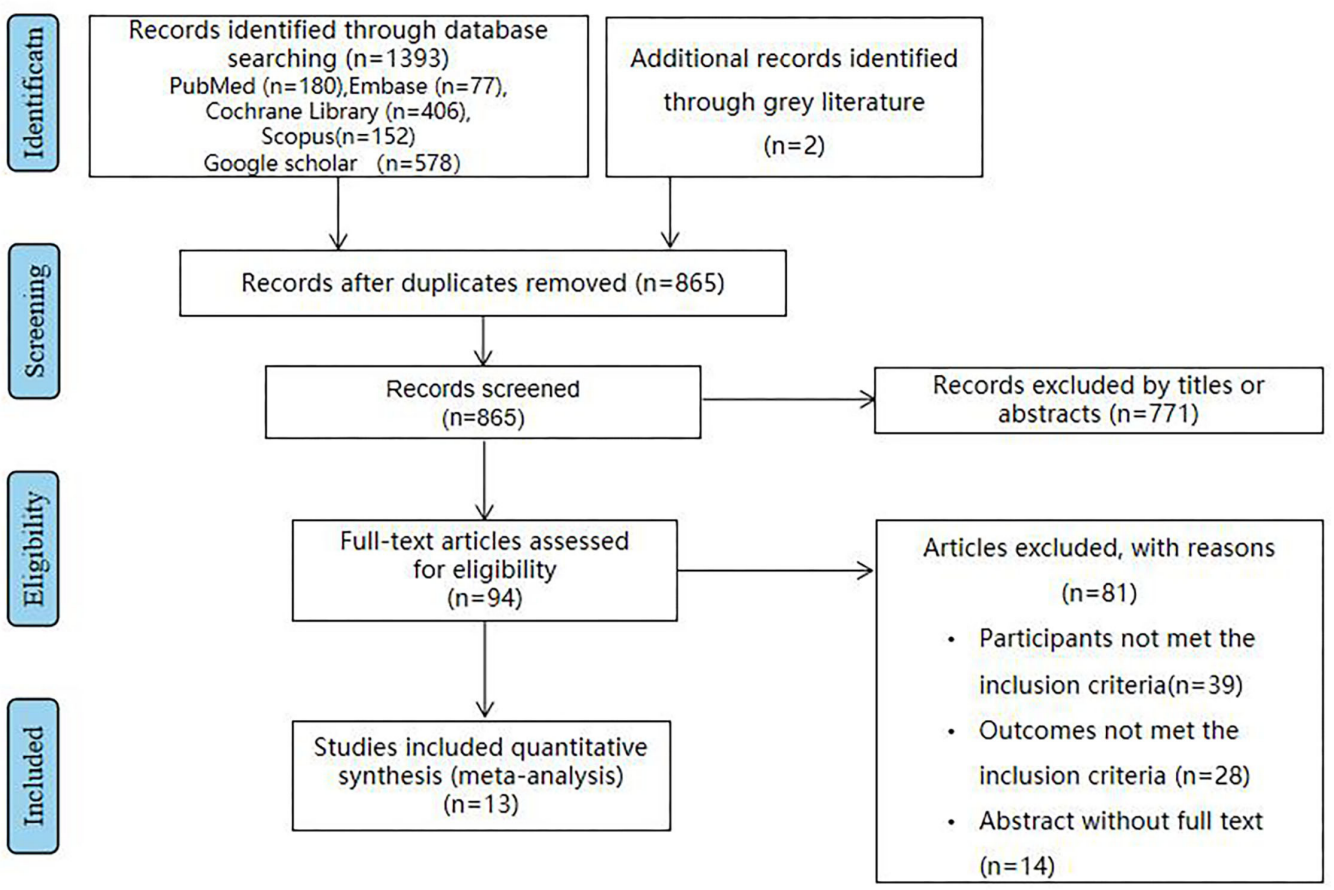
The flowchart shows the article selection process we performed. It shows the process by which relevant studies were retrieved from the databases, assessed, and selected, or excluded. Preferred reporting items for systematic reviews and meta-analyses (PRISMA) diagram for study search was used.

Eventually, 13 articles (14–26) reporting 1,101 patients were enrolled in this study (Figure 1). The baseline characteristics are summarized in Supplementary Table 1. Intervention duration varied from 8 weeks to 6 months. Studies followed different MetS diagnostic criteria, namely, six studies used the Adult Treatment Panel III criteria (17, 18, 20, 21, 24, 26); three studies used National Cholesterol Education Programme/Adult Treatment Panel III (NCEP-ATP III) criteria (14, 15, 19); two studies used the Joint Interim Statement (JIS) (16, 23); and two studies used International Diabetes Federation criteria (IDF) (22, 24). The dietary patterns of the intervention received by the intervention groups were as follows: low-calorie diet, fatty acids, berry, and whole wheat. We selected the following inflammatory markers: IL-1β, IL-6, TNF-α, and CRP for analysis. They were measured by enzyme-linked immunosorbent assay (ELISA) in all the studies.

### Intervention Details

In the included 13 trails, the following six types of dietary intervention patterns were investigated: berry (16, 20, 26), fatty acids (14, 15, 19), low-calorie diet (22, 23), whole wheat (18, 21, 24), whole egg (17), and pistachio nuts (25). The berry intervention patterns included cranberry and black raspberry. Subjects received the same dose of berry diet or placebo. The fatty acids diet intervention patterns referred to diet alone or diet plus omega-3 polyunsaturated fatty acids (PUFAs) supplementation. The low-calorie diet included 50–60% carbohydrate, <30% total fat, and <10% saturated fat. The whole wheat diet included wholegrain or refined cereal products. The whole egg intervention pattern referred to three whole eggs containing 0 g carbohydrate, 16 g protein, and 12 g fat. The pistachio nuts diets referred to participants advised to take pistachios for 20% of total energy.

### Quality of the Included Studies

**Figure 2:**
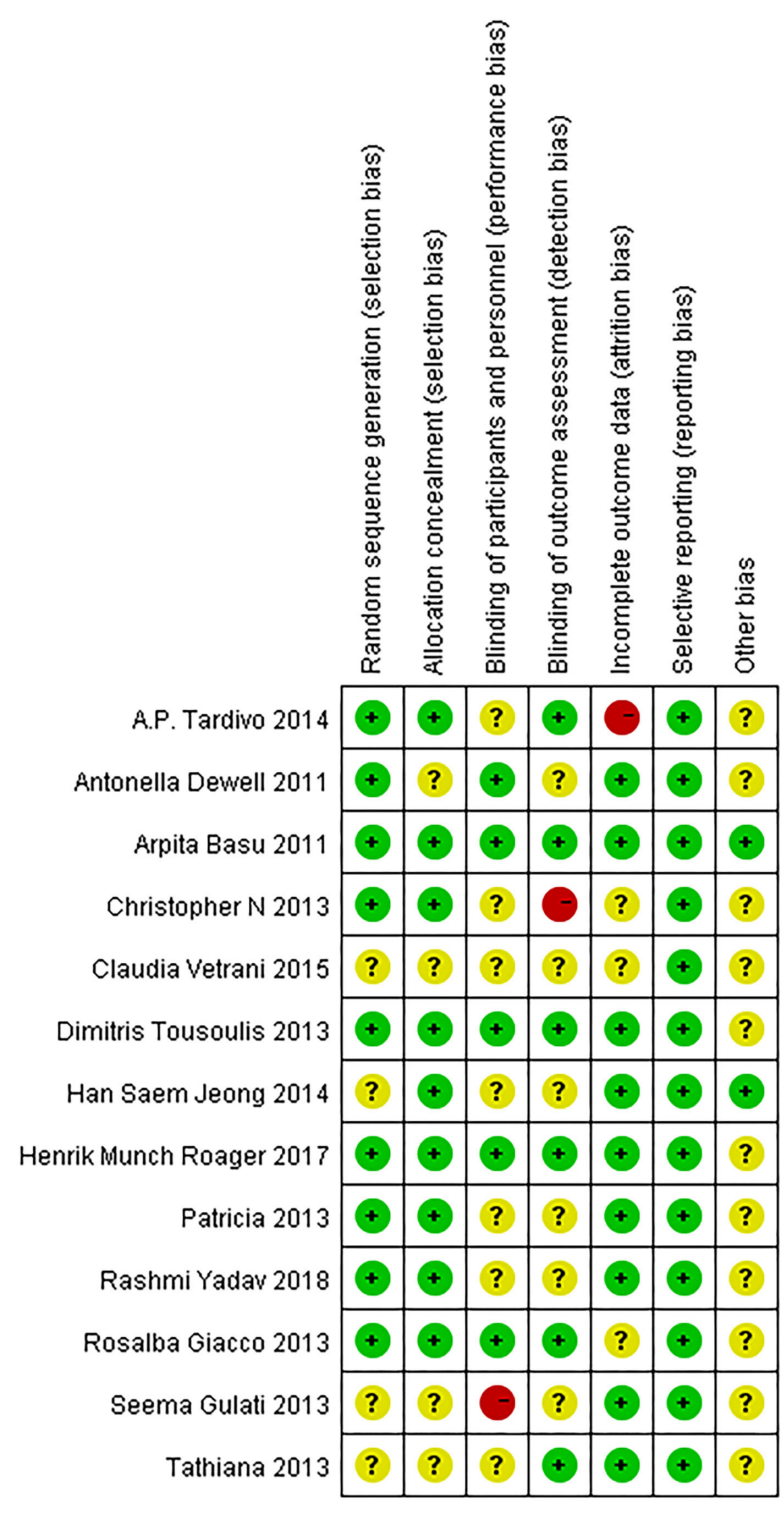
Risk of bias summary.

**Figure 3:**
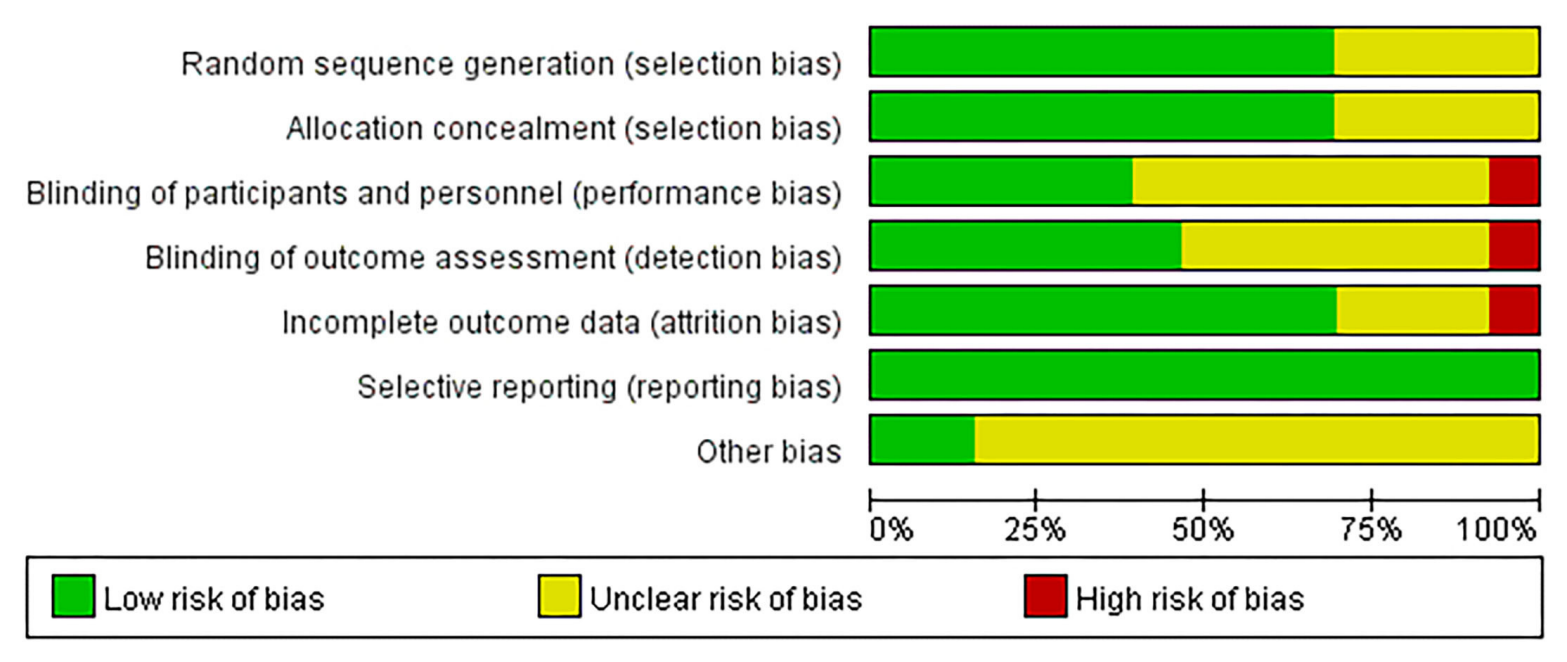
Risk of bias graph.

A risk of bias summary is depicted in Figure 2, and the risk of bias estimation within each of the studies selected is shown in Figure 3. Random sequence generation was adequate in the 5 trials. Three of them have received the maximal score, and none was considered low quality.

### Effect of Diet on IL-1β

**Figure 4:**
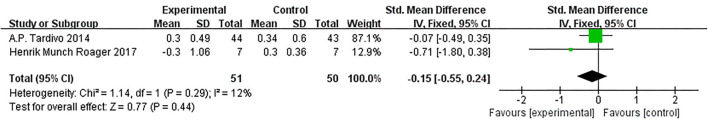
Results of a meta-analysis for the effects of interleukin−1β (IL-1β). Study effect sizes of IL-1β levels differences between metabolic syndrome (MetS) and controls. Each data marker represents a study, and the size of the data marker is proportional to the total number of individuals in that study. The summary effect size for the IL-1β levels of each study is denoted by a diamond. Effect estimates are reported as standardized mean difference (SMD) and 95% confidence intervals. I^2^ represents the magnitude of heterogeneity. *p* ≤ 0.05 is considered as significant.

Based on data from 2 trials (101 participants) (15, 21), we found no effect of diet intervention on IL-1β change compared with control (SMD = −0.08, 95% CI: −0.30, 0.14; Figure 4).

### Effect of Diet on IL-6

**Figure 5:**
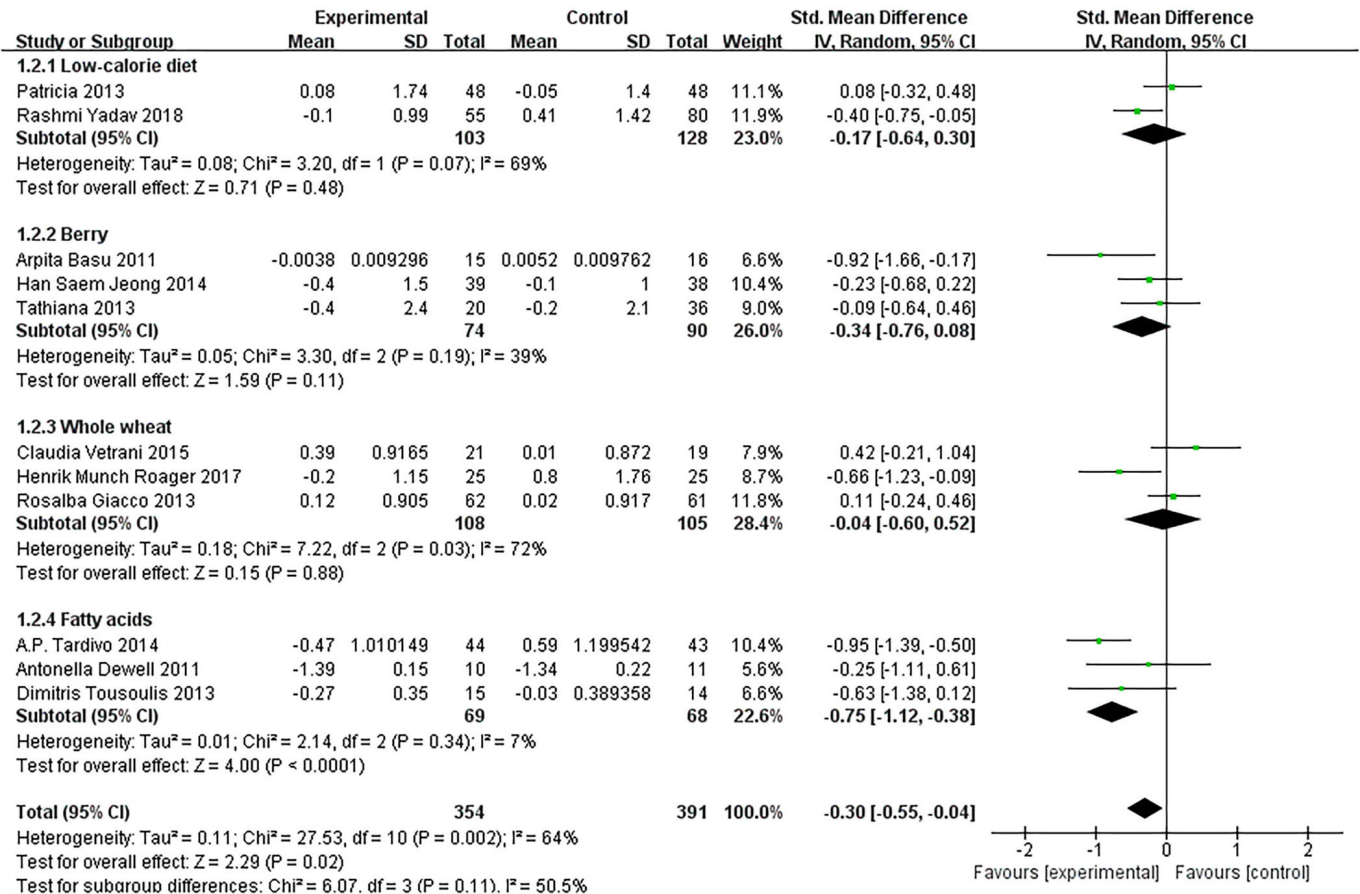
The subgroup analyses for the interleukin-6 (IL-6) levels by dietary intervention. Study effect sizes of the subgroup analyses for the IL-6 levels differences by dietary intervention between MetS and controls.

**Figure 6:**
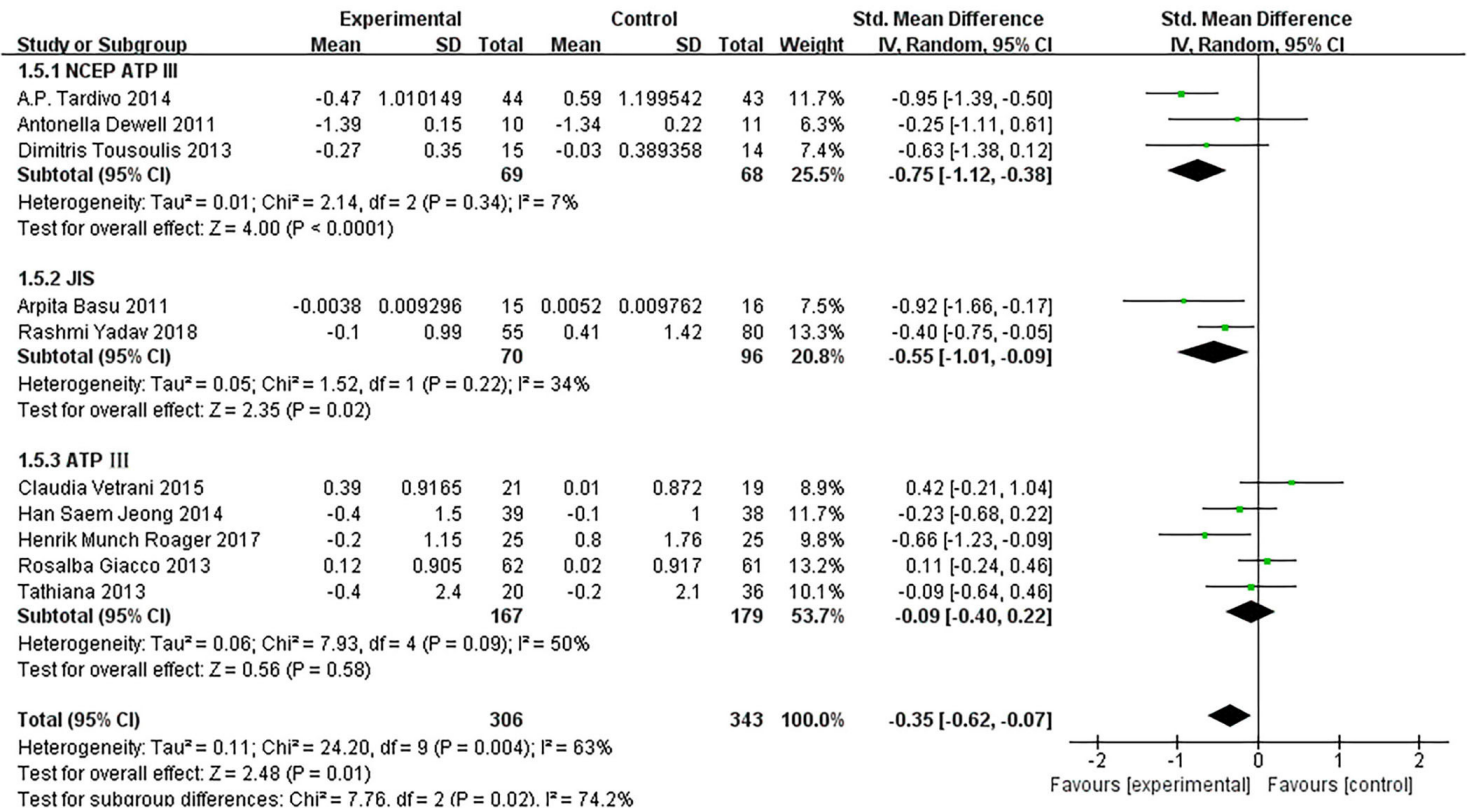
The subgroup analyses for the IL-6 levels by MetS assessment method. Study effect sizes of the subgroup analyses for the IL-6 level differences by MetS assessment method between MetS and controls.

Of the 11 included studies (14–16, 18–25), when all the data were pooled in the meta-analysis, overall IL-6 levels in dietary patients were significantly lower than controls (SMD = −0.30, 95% CI = −0.55, 0.04, *p* = 0.02, I^2^ = 64%). Figure 5 demonstrates the subgroup analyses for the IL-6 levels of between dietary intervention and the controls. The IL-6 of participants who underwent low-calorie diet, berry, whole wheat, and fatty acids dietary intervention decreased compared with the control group (SMD = −0.17, 95% CI = −0.64, 0.30; SMD = −0.34, 95% CI = −0.76, 0.08; SMD = −0.04, 95% CI = −0.60, 0.52; SMD = −0.75, 95% CI = −1.12, −0.38). Figure 6 demonstrates the subgroup analyses by MetS assessment method and shows how the NCEP ATP III and JIS assessment decreased compared with the control group (SMD = −0.75, 95% CI = −1.12, −0.38; SMD = −0.55, 95% CI = −1.01, −0.09).

### Effect of Diet on TNF-α

**Figure 7:**
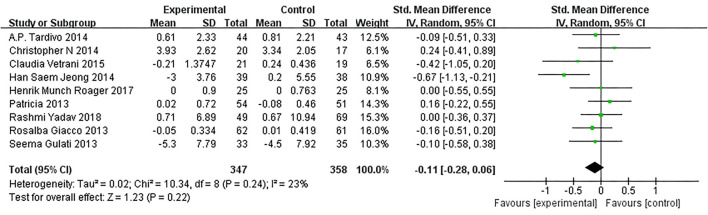
Results of a meta-analysis for the effects of tumor necrosis factor α (TNF-α). Study effect sizes of the subgroup analyses for the TNF-α levels differences between MetS and controls.

**Figure 8:**
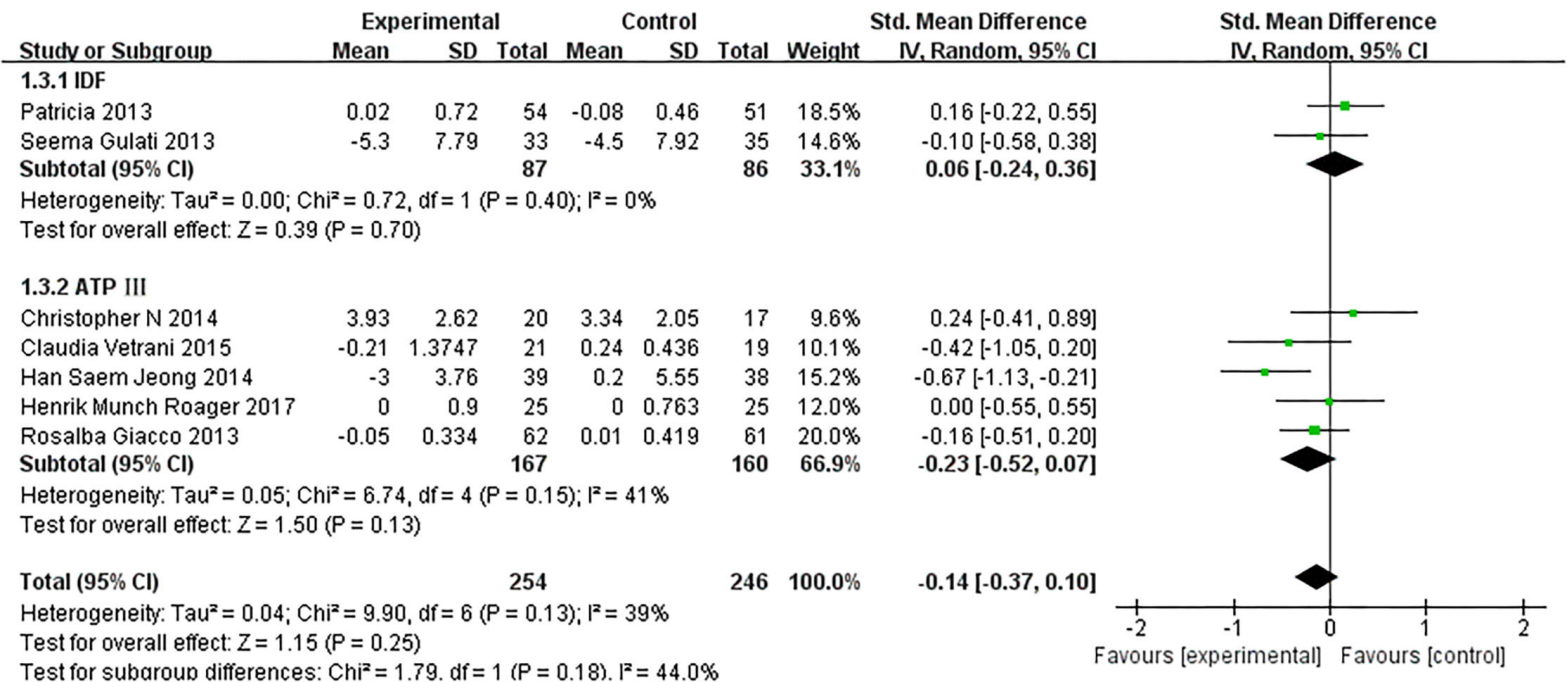
The subgroup analyses for the TNF-α levels by MetS assessment method. Study effect sizes of the subgroup analyses for the TNF-α levels differences by MetS assessment method between MetS and controls.

As presented in Figure 7, our overall pooled analysis did not reveal the association between dietary intervention and the TNF-α levels (15, 17, 18, 20–24, 26) (SMD = −0.11, 95% CI: −0.28, 0.06). According to the assessment method of MetS, the effect estimate did not change considerably (Figure 8).

### Effect of Diet on CRP

**Figure 9:**
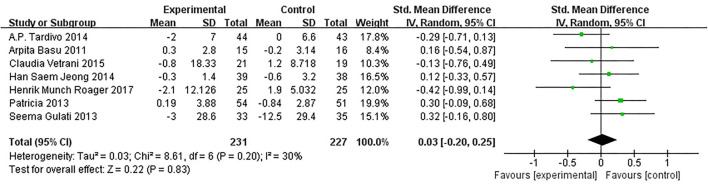
Results of a meta-analysis for the effects of c-reactive protein (CRP). Study effect sizes of the subgroup analyses for the CRP levels differences between MetS and controls.

**Figure 10:**
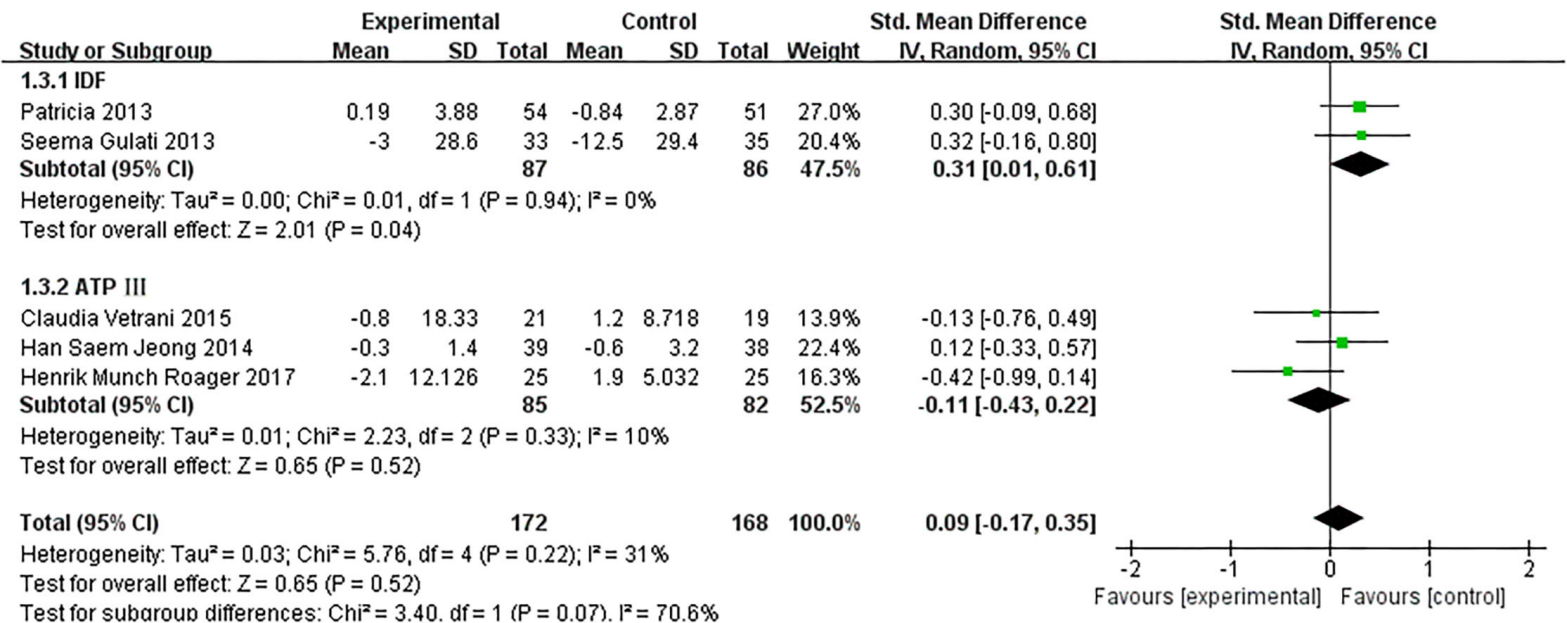
The subgroup analyses for the CRP levels by MetS assessment method. Study effect sizes of the subgroup analyses for the CRP levels differences by MetS assessment method between MetS and controls.

Based on data from 8 trials (458 participants) (15, 16, 18, 20–22, 26), changes on CRP due to dietary intervention were not found compared with control (SMD: 0.03, 95% CI: −0.20, 0.25; Figure 9). Similarly, the subgroup of MetS definitions did not change (Figure 10).

### Publication Bias

The scatter funnel plot in IL-6 levels appeared symmetrical, indicating the absence of publication bias. Additionally, Egger's test detected no publication bias (*p* = 0.320). The number of studies that analyzed IL-1β, TNF-α, and CRP levels was <10. Hence, it was inadequate to perform a publication bias test.

## Discussion

To our knowledge, this is the first meta-analysis to provide evidence that dietary intervention could improve immunological properties, particularly IL-6, in MetS. Further analysis based on subgroups indicated that these results were affected by diet patterns of MetS. Despite this, it did not reveal the association between dietary intervention and the IL-1β, CRP, and TNF-α levels.

Chronic systemic inflammation had long been considered as a major factor in the development and progression of several non-communicable diseases (NCDs), including MetS, diabetes mellitus, obesity, and cancer (27). This was a low-grade variation in the immune homeostasis that adversely regulated metabolic processes over time (28). The occurrence and development of MetS was related to pro-inflammatory cytokines.

Interleukin-1β could increase insulin resistance and promote apoptosis of β cells in animals (29). Although we did not find that dietary intervention could significantly reduce the level of the pro-inflammatory cytokine IL-1β, the anti-IL-1β agents improve insulin secretion and β cell function and reduce inflammation in humans (30).

Interleukin-6 was shown to be a vital mediator of acute phase response with a pleiotropic effect on inflammation during immune response. We found that IL-6 significantly decreased after dietary intervention, especially with fatty acids diet. It may be that dietary intervention can produce immediate changes in MetS, leading to significant changes in IL-6. The JIS and the IDF definitions substantially identify more individuals with MetS than subjects with MetS diagnosed by the NCEP ATP III/ATP III definitions (31). As for the results of subgroup analysis, the NCEP ATP III and JIS assessment show they were statistically significant in identifying patients with MetS, but there was no sufficient evidence.

Tumor necrosis factor-α induced phosphorylation of insulin receptor substrate 1 (IRS-1), preventing insulin from binding to the receptor, which consequently led to insulin resistance (32). In addition, TNF-α and IL-6 mainly came from adipose tissue, which were significantly increased in adults with MetS as they were positively correlated with the degree of obesity (33). Adipose tissue could induce a wide range of acute-phase proteins, such as CRP, fibrinogen, and thrombopoietin, and induce systemic acute phase response (34). These findings supported the role of IL-6, TNF-α, and IL-1β, which are all related to the occurrence of insulin resistance. On the other hand, TNF-α and IL-1β inhibit β Cells and promote their apoptosis (32). Hence, only dietary intervention significantly reduced IL-6 levels, while the rest of the pro-inflammatory cytokines showed a similar trend. We found that these indicators did not reach statistical differences through dietary intervention. This may be due to the patients being in a state of low-grade inflammation. This raises the possibility that data might change significantly if the inclusion standards were to be raised.

Low-calorie diets had been shown to improve the level of inflammatory factors, particularly with advancing age and improving obesity and MetS parameters. However, this was achieved through a significant reduction of total energy intake and supplementing micronutrients (35). The research indicated that after 1 year of low-calorie diet intake, severely obese patients did not show more weight loss, significant changes in MetS indicators, or levels of inflammation markers than those on conventional weight-loss diet (36).

In addition, one of the most commonly used dietary modifications consists of increasing the protein content of the diet. The role of this modification in inflammation was controversial (37). The DiOGenes project reported (apparently for the first time) that dietary protein content influences inflammation, specifically CRP concentrations. This pan-European-controlled dietary intervention study compared a high-protein diet with a low-protein diet in overweight and obese adults and found that the lower protein content appeared to be associated with a further decrease of CRP as compared with the high-protein diet. Excess protein supplementation enhanced phosphorus overload which can lead to acidosis and exacerbated insulin resistance (38). In addition, the meta-analysis by Namazi et al. (39) found no significant association between the pro-inflammatory diet and MetS.

Targeting anti-inflammatory therapies had been applied to patients with MetS. In doing so, the IL1-β inhibitor, Canakinumab, increased insulin secretion (40). In addition, IL-6 inhibitors, tocilizumab, and anti-TNF drugs, were shown to possibly primarily affect insulin-sensitive tissues (41). In the future, the combination of diet and anti-inflammatory drugs may become one of the new approaches to treat MetS.

## Limitation

Firstly, most eligible studies did not adjust potential confounding factors. Secondly, due to the influence of gender, race, ethnicity, and social factors, our findings should be interpreted in different geographic contexts. Thirdly, the reason for the analysis might be due to the limited time of intervention for the inclusion of RCT, leading to no significant changes in inflammatory marker levels. In addition, individual differences, different methods of intervention, and sample size might also directly affect the analysis results. Lastly, according to different diagnostic criteria, long-term trends may have confounded the results and limited generalizability.

## Conclusion

Our study suggests that dietary intervention might decrease IL-6, IL-1β, CRP, and TNF-α in MetS. However, different diets might have different protective mechanisms for MetS. More time and more appropriate dietary patterns are needed to improve the inflammatory state of MetS. In addition, more research is needed to clarify the mechanisms underlying the effect of dietary intervention on this population's inflammatory markers. Anti-inflammatory agents combined with dietary intervention may be therapeutically useful in treating and preventing MetS.

## Data Availability Statement

The original contributions presented in the study are included in the article/Supplementary Material, further inquiries can be directed to the corresponding author/s.

## Author Contributions

ZZ contributed to the study design, wrote the manuscript, reviewed and edited the manuscript. MW, JL, ZZ, HZ, XC, and NW conducted the literature search and performed data extraction and data analysis. MW, JL, and XC did the statistical analyses. MW, JL, XC, XH, QL, and SC contributed to the writing of the manuscript. All authors approved the submitted manuscript.

## Conflict of Interest

The authors declare that the research was conducted in the absence of any commercial or financial relationships that could be construed as a potential conflict of interest.

## Publisher's Note

All claims expressed in this article are solely those of the authors and do not necessarily represent those of their affiliated organizations, or those of the publisher, the editors and the reviewers. Any product that may be evaluated in this article, or claim that may be made by its manufacturer, is not guaranteed or endorsed by the publisher.

## Abbreviations

MetS: metabolic syndrome
IL-1β: interleukin-1β
IL-6: interleukin-6
TNF-α: tumor necrosis factor-α
CRP: C-reactive protein
SMD: Standardized mean difference
CI: Confidence interval
CVD: cardiovascular disease
RCT: Randomized controlled trial
SDs: standard difference
IDF: International Diabetes Federation criteria
JIS: Joint Interim Statement
ATP III: Adult Treatment Panel III criteria
NCEP ATP III: National Cholesterol Education Program, Adult Treatment Panel III
BMI: body mass index
SBP: systolic blood pressure
DBP: diastolic blood pressure
HDL: high-density lipoprotein
LDL: low-density lipoprotein
FPG: Fasting plasma glucose
NR: not reported
ELISA: enzyme-linked immunosorbent assay
PUFAs: polyunsaturated fatty acids
NCDs: non-communicable diseases
IRS-1: insulin receptor substrate 1
DiOGenes: Diet, Obesity, and Genes.
