# Supplementary material for: Effects of Dietary Intervention on Inflammatory Markers in Metabolic Syndrome: A Systematic Review and Meta-Analysis
Source: Front Nutr. 2022 Mar 31;9:846591. doi: 10.3389/fnut.2022.846591 (PMC9008568; doi:10.3389/fnut.2022.846591)
Supplement: Supplementary file 3 [file Table_3.DOCX]

**Supplementary File 3**

**The search strategy of the present systematic review and meta-analysis**

PubMed

1. "Metabolic Syndrome"[Mesh]
2. "Metabolic Syndromes"[Title/Abstract]
3. 1 or 2
4. "Inflammation Mediators"[Mesh]
5. "Mediators, Inflammation"[Title/Abstract]
6. 4 or 5
7. "Interleukin-1beta"[Mesh]
8. "Interleukin 1beta"[Title/Abstract]
9. 7 or 8
10. "Interleukin-6"[Mesh]
11. "Interleukin 6"[Title/Abstract]
12. 10 or 11
13. "Tumor Necrosis Factor-alpha"[Mesh]
14. "Tumor Necrosis Factor alpha"[Title/Abstract]
15. 13 or 14
16. "C-Reactive Protein"[Mesh]
17. "C Reactive Protein"[Title/Abstract]
18. 16 or 17
19. 6 or 9 or12 or 15 or 18
20. "Diet"[Mesh]
21. "Diets"[Title/Abstract]
22. 20 or 21
23. 3 and 19 and 22

**Filters**

Randomized Controlled Trial, from 2011 - 2021

The PubMed strategy and RCT filter was adapted to other database
